# Prevalence of low back pain in emergency settings: a systematic review and meta-analysis

**DOI:** 10.1186/s12891-017-1511-7

**Published:** 2017-04-04

**Authors:** Jordan Edwards, Jill Hayden, Mark Asbridge, Bruce Gregoire, Kirk Magee

**Affiliations:** 1 Department of Epidemiology & Biostatistics, Schulich School of Medicine & Dentistry, Western University, London, ON Canada; 2 Department of Community Health and Epidemiology, Dalhousie University, Halifax, NS Canada; 3 Department of Emergency Medicine, Charles V. Keating Emergency and Trauma Centre, Halifax, NS Canada

## Abstract

**Background:**

Low back pain may be having a significant impact on emergency departments around the world. Research suggests low back pain is one of the leading causes of emergency department visits. However, in the peer-reviewed literature, there has been limited focus on the prevalence and management of back pain in the emergency department setting. The aim of the systematic review was to synthesize evidence about the prevalence of low back pain in emergency settings and explore the impact of study characteristics including type of emergency setting and how the study defined low back pain.

**Methods:**

Studies were identified from PubMed and EMBASE, grey literature search, and other sources. We selected studies that presented prevalence data for adults presenting to an emergency setting with low back pain. Critical appraisal was conducted using a modified tool developed to assess prevalence studies. Meta-analyses and a meta-regression explored the influence of study-level characteristics on prevalence.

**Results:**

We screened 1187 citations and included 21 studies, reported between 2000 and 2016 presenting prevalence data from 12 countries. The pooled prevalence estimate from studies of standard emergency settings was 4.39% (95% CI: 3.67-5.18). Prevalence estimates of the included studies ranged from 0.9% to 17.1% and varied with study definition of low back pain and the type of emergency setting. The overall quality of the evidence was judged to be moderate as there was limited generalizability and high heterogeneity in the results.

**Conclusion:**

This is the first systematic review to examine the prevalence of low back pain in emergency settings. Our results indicate that low back pain is consistently a top presenting complaint and that the prevalence of low back pain varies with definition of low back pain and emergency setting. Clinicians and policy decisions makers should be aware of the potential impact of low back pain in their emergency settings.

**Electronic supplementary material:**

The online version of this article (doi:10.1186/s12891-017-1511-7) contains supplementary material, which is available to authorized users.

## Background

Low back pain is one of the most common and costly forms of musculoskeletal pain [1]. The individual lifetime prevalence of low back pain is approximately 49-90% [2] and approximately 25% of patients presenting for care with low back pain will have another episode within one year [3]. Over the past quarter century there has been an increasing interest in researching the prevalence of low back pain [4]. These estimates are important, as they can serve as a basis for etiologic studies and healthcare evaluation [4].

The majority of patients, who seek care for low back pain, are initially evaluated by a primary care physician [2]. Nevertheless, a governmental report from Canada [5] and research conducted in the US [6] suggest that low back pain is a top five presenting complaint in the emergency department. A comprehensive scoping review of the literature [7] revealed no systematic review on the prevalence of low back pain in the emergency department, though several international studies on the topic were identified [8–12]. There is currently a need to synthesize the available literature and provide prevalence estimates for researchers, health care providers and administrative and policy decision makers around the world [4].

Our objectives in this study were to systematically identify and synthesize available studies of prevalence of low back pain in the emergency department. We explored heterogeneity by comparing prevalence estimates for types of emergency department settings and for different definitions of low back pain used in included studies.

## Methods

### Search strategy

We searched electronic databases PubMed and EMBASE (to May 2016) using controlled vocabulary and keyword variations of the concepts: emergency department, low back pain and prevalence (see Additional file 1 and Additional file 2). We conducted citation searches of seminal studies [9, 10, 12–15]. For studies with greater than 500 citations, we searched within citations for “emergency department” using Google Scholar. We reviewed reference lists of included studies to identify other potentially relevant studies. Additionally, our literature search incorporated all relevant literature that was identified in a broad scoping review mapping published research studies about back pain in the emergency department [7]. We searched for relevant subsequent publications for any abstracts identified.

We searched the grey literature guided by the ‘Grey Matters’ checklist [16]; we searched all websites listed in the checklist under the headings of health economics [e.g. Public Health Agency of Canada] or health statistics [e.g. Canadian Institute for Health Information and the CDC National Centre for Health Statistics], excluding pharmacological based websites (see Additional file 3). Websites that we reviewed collected data from Canada, the United States, Australia, Ireland, England, Scotland and five international databases (e.g. World Health Organization). We searched these websites 10 pages deep using the following search criteria, “low back pain” and “prevalence” and “emergency department”. We did not restrict searches by language or date. The grey literature search was conducted in May 2016.

### Selection criteria

We included studies that investigated patients presenting to emergency settings. We defined ‘emergency setting’ as all pre-hospital, emergency, ambulatory, outpatient, accident, trauma, triage and urgent care services. Standard emergency settings provide initial treatment to patients with a broad spectrum of illnesses and injuries, some of which may be life threatening and require immediate action. For completeness, we included non-standard emergency settings, which provide care for a limited population and/or limited spectrum of illness and injuries (for example, orthopedic emergency settings). Additionally, we included studies from any year, written in any language.

We classified emergency settings by size. Emergency department settings with less than 10,000 annual visits were categorized as ‘rural’, those with more than 10 000 annual visits were categorized as ‘metropolitan’ and we separately considered studies that used nationally representative samples of emergency settings.

We categorized emergency settings by country level health care system funding. Studies were classified as being either using primarily a public funding system or a private funding system. If information was not provided in the publication, this data was collected from governmental websites and online encyclopedias identified using the search engine Google. We defined publicly funded healthcare systems as systems with no out of pocket costs associated with care in an emergency setting. We defined private funded healthcare systems as systems that require out of pocket payments for most visits to emergency settings and many procedures.

We included studies that measured adults presenting with low back pain. We defined adults as individuals over the age of 14, as this is an age where patients are likely to be diagnosed and treated as an adult [17]. If study selection criteria were mixed or unclear, we defined studies with an adequately ‘adult’ population as those with a minimum mean age of 30 years.

We included studies that used any definition of back pain. We used subgroups to explore the impact of study definitions of low back pain. We categorized studies that identified patients from presenting complaint codes and studies that captured their study population from diagnostic codes, and we collected information on the specific coding system used.

We categorized low back pain definitions as ‘broad’ or ‘narrow’. Studies were defined as ‘broad’ if they used a general definition of ‘back pain’ to define their prevalence estimate. These studies may have included some individuals with back pain in regions other than the low back (for example, thoracic spine). Studies were defined as ‘narrow’ if they used the definition of ‘low back pain’ or ‘non-specific low back pain’, or were limited to pain complaints in the lumbar region.

We included studies that presented data about the prevalence, including presentation of a prevalence rate (total number of adults presenting to an emergency setting with low back pain / total number of individuals presenting to the emergency setting over a specified period of time), or raw data to allow prevalence calculation.

### Study selection and data collection

Two independent reviewers screened the titles and abstracts from the electronic database searches for studies meeting our selection criteria. In the case of disagreement, resolution was achieved by discussion with a third reviewer. The primary author screened the titles from the grey literature searches, reference lists (from included studies), results of the scoping review [7], and citation searches. Full articles were obtained for potentially relevant studies, or where relevance was unclear; two authors independently assessed the full text to determine eligibility prior to data extraction.

Two independent reviewers performed data extraction. In the case of disagreement, resolution was achieved by including a third reviewer. We used a data extraction form (see Additional file 4), to record information about the methods and results of each included study, including study objectives, location and type of emergency setting, study period, sample size, the definition of low back pain used by the study authors to calculate prevalence, population characteristics including age and sex, and the prevalence estimate. In studies using the same datasets, we extracted the prevalence data of the study that was conducted over the longest period of time and rated as having the lowest risk of bias. Finally, one reviewer collected information from an independent Google search to characterize each study’s country-level healthcare system funding system.

### Critical appraisal

Two independent reviewers critically appraised each included study using a tool developed by Hoy et al., [3], to assess prevalence studies (see Additional file 5) [18]. In the case of disagreement or uncertainty, discussion was used to reach consensus with a third reviewer. The modified tool assesses each study according to nine domains: three external validity domains, and six internal validity domains, plus one item assessing overall risk of bias. The external validity domains assess the target population; sampling and non-response bias, while the internal risk of bias domains assess data collection, case definitions, assessment tools, prevalence period and an assessment of the numerator and denominator. We modified the tool by omitting an additional domain that assesses whether the study population represents the national population, which was not relevant to our review. The reviewers rated each of the nine domains as either high or low risk of bias; the overall risk of bias was rated as low, moderate or high risk of bias. We judged an overall low risk of bias if a study scored ‘low risk of bias’ on all domains. A moderate risk of bias study had one to two domains rated as a high risk of bias, and an overall high risk of bias study had three or more domains rated as a high risk of bias.

### Analysis

Descriptive analyses were used to report study characteristics. We reported prevalence ranges, information on emergency settings, study methodology, and study populations.

#### Meta-analyses

We used meta-analyses to pool prevalence estimates for sufficiently homogeneous groups of studies conducted in standard emergency settings. Subgroup analyses explored the impact of study level characteristics: back pain definition, coding system used for definitions of low back pain, health care system and emergency setting on prevalence estimates. This is an essential part of analyzing prevalence studies, as the largest contributor to heterogeneity is most likely due to differences in the way the studies were carried out [19]. We were interested in further subgroup analyses (e.g., rural settings), however, we were limited by the available literature.

For all meta-analyses, we used a random-effects model to calculate mean prevalence rates and 95% confidence intervals. In this model, larger studies have more narrow confidence intervals and higher weight on the pooled estimate. We normalized the distribution of the prevalence rates by transforming the prevalence estimates reported in the publications (or calculated using reported data) using a double arcsine transformation. This transformation addresses the main issues associated with performing meta-analysis of prevalence estimates. It stabilizes the variance when pooling prevalence estimates and reduces the bias when combining prevalence estimates close to 0 or 100 [19]. The rates were restored for presentation of results. We assessed statistical heterogeneity using the Q statistic and I^2^ index [20]. We used forest plots to graphically present prevalence estimates and 95% CIs. We tested subgroups for inter-group heterogeneity using the Q statistic [21].

#### Meta-regression

We performed a random effects meta-regression analysis to explore the independent association of three clinically relevant characteristics with prevalence: the coding system used for definitions of low back pain, health care system funding, and study risk of bias [22]. Results of the analysis were used to determine the variance explained by the covariates and their contribution to the total variance in the prevalence estimates. For our analysis we used the Knapp-Hartung variance estimator and associated *t*-test to calculate p-values and confidence intervals [23]. We performed sensitivity analyses by excluding studies judged to have a high risk of bias. All analyses were performed using STATA 13.1

#### Assessing the quality of evidence (GRADE)

We adapted components of the GRADE [24] framework to assess the overall quality of the available evidence on the prevalence of low back pain in the emergency setting, judged as high, moderate, low or very low quality evidence based on: study limitations (overall risk of bias of the evidence identified), imprecision (study sample sizes), indirectness (generalizability of included studies) and inconsistency (unexplained heterogeneity) [17, 24]. Additional file 6 provides additional detail about our assessment of the overall quality of the evidence.

## Results

**Fig. 1:**
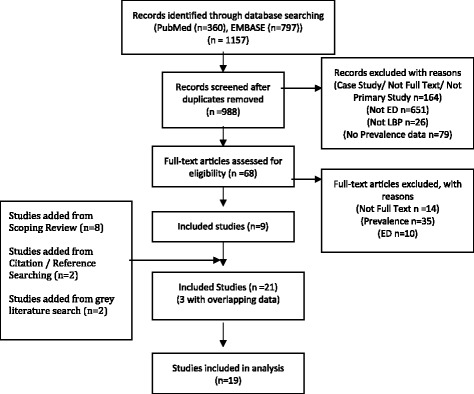
Flow chart of the selection of studies to be included in our systematic review

Our search of electronic databases identified 1187 citations; we screened 68 full texts and included nine studies from the electronic search. We identified an additional twelve studies from alternative search strategies for a total of 21 relevant publications, 3 of which used overlapping data (Fig. 1).

### Study characteristics

The 21 publications, reporting on 19 studies, were conducted between 2000 and 2016 from 12 countries. Included studies used a variety of data sources, including administrative databases, surveys and patient charts [8–10, 12–15, 25–37] (Table 1). Fifteen studies collected data retrospectively. Sixteen studies collected data from standard emergency settings, one study was conducted in an orthopaedic emergency department [15], one study was conducted in medical presidiums following an earthquake [26], and one study collected data from emergency ambulance calls [33]. Reported prevalence estimates of low back pain in the emergency setting ranged from 0.9% to 17%.

**Table 1:** Methods and results of included studies

Study	Location of study	Duration of data collection	ED Setting	Health Care System Funding	Age (Mean/ Median)	Sex (%Fem)	Definition of LBP	Coding System,	Sample Size	Prevalence Estimate
Angeletti, 2013	Italy	1-12 months	Non-Standard ED: Presidium (Post Seismic Period)	Private	53^c^	36^d^	Narrow	Diagnosis-Patient Charts	958	4.9%
Astete, 2016	Spain	1-5 Years	Standard ED: Metropolitan	Public	57	61	Narrow	Diagnosis	2000^g^	2.9%
Cordell, 2002	USA	<Month	Standard ED: Metropolitan	Private	30	55	Narrow	Complaint	1665	7.6%
Dutch, 2008	Australia	1-5 Years	3 Standard EDs: Metropolitan	Private	>18	-	Broad	Complaint	104 705^g^	3.3%
Fialho, 2011	Brazil	<Month	Standard ED: Metropolitan	Public	41^c^	58^d^	Narrow	Complaint- Patient Charts	392	8.4%
Hoppe, 2013	USA	1-12 Months	Standard ED: Metropolitan	Private	38	49	Broad	Complaint-EDIS	300^7^	5.4%
Kao, 2014^a^	USA	≥5 Years	Standard ED: National Representation	Private	44	45	Broad	Diagnosis-ICD-9^f^	323 186- 1.1 Billion	3.99%
Friedman, 2010^a^	USA	≥5 Years	Standard ED: National Representation	Private	40	51	Narrow^e^	Diagnosis-ICD-9^f^	183 64 3 -114 million	2.3%
Niska, 2010^a^	USA	1-5 Years	Standard ED: National Representation	Private	>18	47	Narrow	Diagnosis-ICD-9^f^	39 393 000	1.9%
Lovegrove, 2011	Australia	1-5 Years	Standard ED: Metropolitan (All EDs in Perth)	Private	46^c^	51^d^	Broad	Complaint-EDIS	1 171 731	1.9%
Mapoure, 2015	Cameroon	≥5 Years	Standard ED: Metropolitan	Public	40	51	Narrow^e^	Diagnosis-ICD-9^f^	183 633	2.3%
Marinos, 2008	Greece	1-5 Years	Non-Standard ED: Orthopedic	Private	>18	47	Narrow	Diagnosis-ICD-9	39 172	17.1%
NHAMCS, 2010	USA	1-5 Years	Standard ED: National Representation	Private	37^b^	57	Narrow	Diagnosis-ICD-9	84 886 000	3.7%
Owens, 2008	USA	1-5 Years	Standard ED: National Representation	Private	47	57	Broad	Complaint-ICD-9^f^	128 350 000	5.8%
Philips 2012	Barbados	1-12 Months	Non-Standard ED: Emergency Calls	Private	-	-	Broad	Complaint	8875	0.9%
Ross, 2003	USA	≥5 Years	Standard ED: Metropolitan Observation Unit	Private	53	59	Broad	Diagnosis	22530	4.9%
Silman, 2000	UK	1-12 months	2 Standard EDs: Metropolitan	Public	>18	-	Narrow	Complaint-Survey	A: 5147 B: 1459	A: 3.2% B: 4.7% (Pooled 3.5)
Tcherny-Lessenot, 2003	France	<Month	Standard ED: Metropolitan	Public	37	50	Broad	Complaint-Survey	729^g^	10.8%
Thiruga-nasamban-damoorthy, 2014	Canada	1-12 months	2 Standard EDs: Metropolitan	Public	49^c^	51^d^	Narrow	Diagnosis-ICD-10^f^	31705	2.2%
Waterman, 2012	USA	≥5 Years	Standard ED: National Representation	Private	39^c^	49^d^	Narrow	Diagnosis-ICD-9^f^	1 820 000	3.15%
Yan, 2015	Cambodia	1-12 months	2 Standard EDs: Metropolitan	Private	42	64	Broad	Complaint	1295	5.6%

Notes: “Definition of LBP”, “Narrow” indicates studies using narrow definitions of low back pain. They used a definition of ‘low back pain’ or ‘non-specific low back pain’, or were limited to pain complaints in the lumbar region, while “Broad” indicates studies using broad definitions of low back pain. They used a general definition of ‘back pain’ to define their prevalence estimate, which may have included some individuals with back pain in regions other than the low back pain (for example, thoracic spine). “Coding System”. “Complaint” indicates studies using presenting complaints for their definitions of low back pain while “Diagnosis” indicates studies using diagnosis codes for their definition. ^a^: Indicates that studies used the same database. ^b^: Had to calculate age using age ranges. ^c^: indicates that the age calculation was derived from the back pain population, not the entire presenting population*, (>18)* indicates that the study collected data from an adult population 18+. ^d^: indicates that the % female calculation was derived from the back pain population, as opposed to the entire presenting population to the ED. ^e^: *Complaint,* patients presenting with a complaint of back pain to the ED, *Diagnosis,* patients diagnosed with back pain in the ED. 5 = NON-SPECIFIC. ^f^: indicates that the study used and presented specific codes for their definitions of LBP. ^g^: *Astete:* random sample chosen from all patients presenting to the ED, *Dutch:* excluded younger than 18, left before being seen by a physician, dead on arrival and trauma patients. *Tcherny-Lessenot:* Survey data, missing some individuals who could not fill out survey or too many presenting to the ED at one time, *Hoppe:* Individuals were receiving opioid in the population they used to explore back pain, *Thirun:* Used age and sex for patients with serious pathology

Four included studies were judged to have high risk of bias, 11 with moderate risk of bias, and four with low risk of bias (Table 2). Studies with high overall risk of bias inconsistently defined low back pain, didn’t use coding systems for their definitions of low back pain, and had prevalence data that was collected over less than one year. Further details on studies with moderate and high risk of bias can be found in (Table 2).

**Table 2:** Risk of bias analysis for all studies included in the review. Hoy et al., 2012, developed this risk of bias tool.

Authors	Sampling frame represent target population?	Random selection for sample or census?	Likelihood of nonresponse bias minimal?	Data collected directly from subjects?	Acceptable case definition?	Study Instrument has validity and reliability?	Same mode of data collection for all subjects?	Length of prevalence period appropriate?	Appropriate numerator and denominator?	Overall Risk Assessment
Angeletti	L	L	L	L	L	H	L	H	L	Mod
Astete	L	L	L	L	L	H	L	L	L	Mod
Cordell	L	L	L	L	L	H	L	H	L	Mod
Dutch	L	L	L	L	H	H	L	L	L	Mod
Fialho	L	L	L	L	L	H	L	H	L	Mod
Hoppe	L	L	L	L	H	H	L	H	L	High
Kao^a^	L	L	L	L	L	L	L	L	L	Low
Friedman^a^	L	L	L	L	L	L	L	L	L	Low
Niska^a^	L	L	L	L	L	H	L	L	L	Mod
Lovegrove	L	L	L	L	L	L	L	L	L	Low
Mapoure	L	L	L	L	L	H	L	H	L	Mod
Marinos	L	L	L	L	H	H	L	L	L	Mod
NHAMCS	L	L	L	L	L	H	L	L	L	Mod
Owens	L	L	L	L	L	L	L	L	L	Low
Philips	L	L	L	L	H	H	L	L	L	Mod
Ross	L	L	L	L	H	H	L	L	L	Mod
Silman	L	H	H	L	L	H	L	H	L	High
Tcherny-Lessenot	H	H	H	L	H	H	L	H	L	High
Thiruga-nasamban-damoorthy	L	L	L	L	L	L	L	H	L	Mod
Waterman	L	L	L	L	L	L	L	L	L	Low
Yan	L	L	L	L	H	H	L	H	L	High

^a^: Indicates studies using the same database

### Analyses

**Fig. 2:**
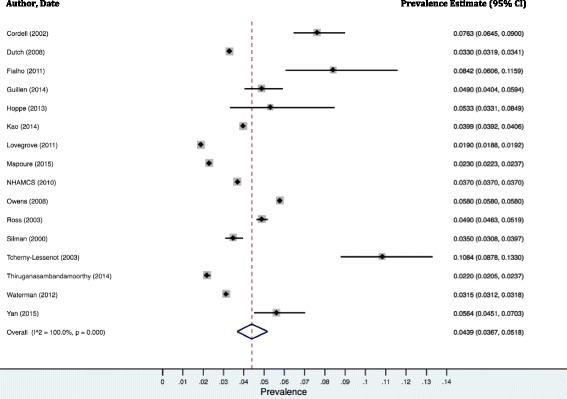
Random effects meta-analyses of prevalence estimates from included studies with standard emergency settings (*n* = 16)

The pooled prevalence estimate for standard emergency settings was 4.39% (3.67%-5.18%). There is significant heterogeneity in the results of this analysis, as assessed by the Q statistic (5.9e + 05, *p* = 0.00) and the I^2^ index (100.0%) (Fig. 2).

**Fig. 3:**
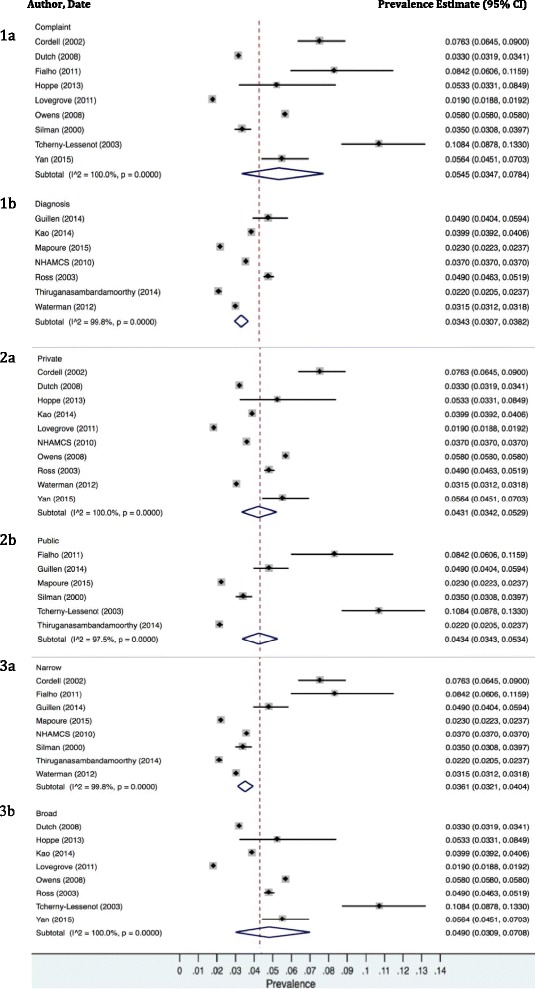
Random effects meta-analyses of prevalence estimates from included studies with standard emergency settings (*n* = 16). The pooled estimate (*red line*) is representative of the 16 studies included in each subgroup. Studies are grouped by the approach used to define the definition of low back pain: Meta-analysis 1 – Studies grouped by coding system used for the definition of low back pain, 1a “Complaint” indicates studies using presenting complaints for their definitions of low back pain, 1b “Diagnosis” indicates studies using diagnosis codes for their definition. Meta analysis 2- Studies are grouped by healthcare system funding, 2a “Private” indicates studies conducted in regions with private healthcare funding. 2b “Public” indicates studies conducted in regions with public healthcare funding. Meta analysis 3- Studies are grouped by definition of low back pain, 3a “Narrow” indicates studies using narrow definitions of low back pain. They used a definition of ‘low back pain’ or ‘non-specific low back pain’, or were limited to pain complaints in the lumbar region. 3b “Broad” indicates studies using broad definitions of low back pain. They used a general definition of ‘back pain’ to define their prevalence estimate, which may have included some individuals with back pain in regions other than the low back pain (for example, thoracic spine)

Subgroup analysis results are presented in (Table 3, Fig. 3). We found that studies using presenting complaints to measure prevalence had a higher pooled prevalence estimate 5.5% (3.5%-7.8%) than studies using diagnosis coding 3.4% (3.1%-3.8%), with significant inter-group heterogeneity *p* = 0.046. None of the covariates investigated in our meta-regression analyses were significantly associated with prevalence estimates (Table 4).

**Table 3:** Subgroup analyses presenting pooled prevalence estimates for various subgroups with and without sensitivity analyses

Subgroup	Category	Arcsine prevalence, 95% CI	Inter-group heterogeneity	Sensitivity analysis, 95% CI (Arcsine Excluding High ROB),	Inter-group heterogeneity
Back Pain Definition	Broad	4.9% (3.1-7.1)	*p* = 0.1850	3.9% (1.9-6.4)	*p* = 0.8574
	Narrow	3.6% (3.2-4.0)		3.6% (3.2-4.1)	
Coding	Complaint	5.5% (3.5-7.8)	*p* = 0.0459	5.0% (2.6-8.2)	*p* = 0.2289
	Diagnosis	3.4% (3.1-3.8)		3.4% (3.1-3.8)	
Health System	Private	4.3% (3.4-5.3)	*p* = 0.9464	4.1% (3.2-5.2)	*p* = 0.2106
	Public	4.4% (3.4-5.3)		3.3% (2.6-4.1)	
Emergency Setting	Standard	4.4% (3.7-5.2)	*p* = 0.7734	4.0% (3.2-4.9)	*p* = 0.7149
	Non-Standard	6.1% (0.0-2.3)		6.1% (0.00-23.2)	

Notes: Back Pain Definition; “Narrow” indicates studies using narrow definitions of low back pain. They used a definition of ‘low back pain’ or ‘non-specific low back pain’, or were limited to pain complaints in the lumbar region, while “Broad” indicates studies using broad definitions of low back pain. They used a general definition of ‘back pain’ to define their prevalence estimate, which may have included some individuals with back pain in regions other than the low back pain (for example, thoracic spine). Coding System; “Complaint” indicates studies using presenting complaints for their definitions of low back pain while “Diagnosis” indicates studies using diagnosis codes for their definition. Health System; “Private” indicates studies conducted in regions with private healthcare funding and “Public” indicates studies conducted in regions with public healthcare funding. Emergency Setting; “Standard” indicates studies provide initial treatment to patients with a broad spectrum of illness and injuries, while “Non-Standard” indicates settings, which provide care for a limited population and/or limited spectrum of illness and injuries

**Table 4:** Meta regression analysis

Covariate	Coefficient	95% Confidence interval		*P*-value
Coding	-0.01989	-0.04472	0.00493	0.106
ROB	0.01032	-0.02085	0.04149	0.485
Health System	0.00309	-0.02580	0.03199	0.819

Notes: “Coding” includes studies grouped by coding system used for the definition of low back pain. In our analysis 1 was given to studies using diagnosis codes for their definition, while 0 was given to studies using presenting complaints for their definition. “ROB” includes studies grouped by risk of bias. In our analysis 1 was given to studies judged to have a moderate to high risk of bias and 0 was given to studies judged to have a low risk of bias. “Health System” includes studies grouped by healthcare system funding. In our analysis 1 was given to studies conducted in regions with private healthcare funding and 0 was given to studies conducted in regions with public healthcare funding

When studies with a high risk of bias were eliminated from our analysis of standard emergency settings, the pooled prevalence estimate was 4% (3.2%-4.9%). Additionally, there was no longer significant inter-group heterogeneity in studies using presenting complaints and studies using diagnosis codes (*p* = 0.229). Other results from our sensitivity analysis can be found in (Table 3).

### Quality of the evidence – GRADE

We judged the overall quality of the evidence available to be moderate and judged that further research could modify our estimate of low back pain in emergency settings. Our judgment was downgraded as we included four studies with high risk of bias and additionally 11 studies with moderate risk of bias. There was a large amount of variability in the prevalence estimates of included studies, and there was a lack of prevalence estimates from some important settings, including rural and from developing nations.

## Discussion

This review provides the first comprehensive search and synthesis of the international literature on the prevalence of low back pain in emergency settings. The result of our synthesis of all prevalence estimates for adults presenting with low back pain to standard emergency settings was 4.39% (3.67%-5.18%). Our pooled estimate indicates that low back pain is a common presenting complaint in emergency settings in our analysis. To provide this perspective, a national trends analysis performed in the US showed that presenting complaints with prevalence of 3.7% (or higher) made up the top 10 presenting complaints in the average American emergency department. Our result is similar to the prevalence of “shortness of breath” (4%) and “fever and chills” (4.4%). For comparison, the highest most common presenting complaint, “any injury”, had a prevalence of 18.2% and the second highest estimate, “cough, upper respiratory or ears/nose/throat symptoms”, had a prevalence of 9.2% [6].

Significant heterogeneity was found in prevalence estimates of the included studies. Prevalence estimates of included studies ranged from 0.9% to 17.1%. Although variation in estimates from different emergency settings were expected, it is important to explore potential sources of heterogeneity, including the types of emergency settings. Though the majority of included studies were conducted in standard emergency settings, there were three studies from non-standard settings, which contributed to the large range of prevalence estimates. For example, the study with the highest prevalence estimate (17%) gathered data from an orthopedic emergency department, where one might expect to find a higher prevalence of low back pain patients [15].

We explored potential sources of heterogeneity by conducting subgroup and meta-regression analyses. Subgroup analyses exploring the impact of study-level characteristics on prevalence estimates found that studies using ‘presenting complaints’ to define low back pain cases were associated with a higher prevalence estimate 5.5% (3.5-7.8) than studies that used diagnostic coding 3.4% (3.1-3.8). This may be due to the fact that prevalence estimates from studies using presenting complaints reflect the symptom of back pain as a chief complaint, which may or may not be caused by the underlying etiology associated with a diagnosis of back pain. Conversely, diagnostic codes represent a specific category of low back pain (for example, non-specific low back pain). We did not find any meaningful results from our meta-regression analysis. This may be due to the small number of studies and many sources of heterogeneity. Additionally, we did not find a meaningful difference in the pooled estimate, once studies with high risk of bias were eliminated. This finding increased our confidence in our overall pooled prevalence estimate.

### Strengths and limitations of our review

A strength of the review was our approach to analysis, which included a meta-analysis, meta-regression, subgroup analysis and sensitivity analyses. These analyses allowed us to explore the effects of study level characteristics on prevalence estimates and test the robustness of our analysis.

Another strength of the study was our use of alternative search strategies, such as the results from a scoping review of back pain in emergency settings. This was important as only 43% of included studies came from our electronic search. We believe this is a result of the poor indexing of prevalence studies in electronic databases rather than an issue with publication bias. We believe future research would benefit if studies were properly indexed with “prevalence” in electronic databases PubMed and EMBASE.

Our study should be considered in the context of the limitations of the evidence available. There are limitations in the generalizability of our results. In our search, we found no studies analyzing the prevalence of low back pain primarily in rural emergency settings. Also, we found no results from developing nations, though these and rural settings may represent unique populations and distinct prevalence rates. Furthermore, our results may not be generalizable to older populations of individuals, as included studies had relatively young mean ages between 30 and 53.

Our exploratory analyses require cautious interpretation. Our pooled estimates are useful to provide context and compare study level characteristics, however, they must be carefully interpreted. Decision makers and clinicians should consider individually relevant emergency settings and applicable study methodologies.

Researchers in the field should concentrate on improving the quality of prevalence estimates for low back pain in emergency settings. This could be achieved by conducting studies that use well defined, transparent, definitions of low back pain e.g. studies using specific triage codes or specific diagnosis codes. Also, there should be an increase in prevalence estimates from rural emergency settings and estimates from developing nations as they may represent unique populations with various low back pain needs.

## Conclusion

This is the first systematic review to explore the prevalence of low back pain in emergency settings. Determining the prevalence is a crucial step in understanding the impact of low back pain in various emergency settings. Our results not only indicate that low back pain is consistently a top presenting complaint, they also reveal that the prevalence of low back pain varies with definition of low back pain and emergency setting. The overall quality of the evidence was judged to be moderate as there was limited generalizability and high heterogeneity in the results. Clinicians and policy decisions makers should be aware of the potential impact of low back pain in their emergency settings. This review will facilitate this discussion and provide context. This review may additionally be used to inform future research, which will allow for more meaningful comparisons between and within emergency settings.

## Additional files

Additional file 1: PUBMED search strategy. (DOCX 87 kb)

Additional file 2: EMBASE Search Strategy. (DOCX 79 kb)

Additional file 3: Grey Literature Search Strategy. (DOCX 54 kb)

Additional file 4: Data Extraction Form. (DOCX 107 kb)

Additional file 5: Risk of Bias Tool Developed By Hoy et al., [3]. (DOCX 69 kb)

Additional file 6: GRADE Concepts Developed by Guyatte et al., 2011. (DOCX 75 kb)
