# Supplementary material for: Prevalence of low back pain in emergency settings: a systematic review and meta-analysis
Source: BMC Musculoskelet Disord. 2017 Apr 4;18:143. doi: 10.1186/s12891-017-1511-7 (PMC5379602; doi:10.1186/s12891-017-1511-7)
Supplement: Supplementary file 1 — PUBMED search strategy. (DOCX 87 kb) [file 12891_2017_1511_MOESM1_ESM.docx]

**Additional File 1: PUBMED Search Strategy**

| emergency medical services[Mesh] | 98220 |
| --- | --- |
| - emergency medicine[Mesh] | 10124 |
| - emergency medicine*[tw] | 15880 |
| - emergency centre*[tw] | 116 |
| - emergency clinic*[tw] | 480 |
| - emergency service*[tw] | 49709 |
| - emergency department*[tw] | 52517 |
| emergency room*[tw] | 13229 |
| emergency ward*[tw] | 856 |
| - emergency unit*[tw] | 1581 |
| - emergency treatment*[tw] | 11753 |
| - emergency care*[tw] | 5907 |
| - emergency patient*[tw] | 1231 |
| - emergency physician*[tw] | 6423 |
| - ambulatory care[Mesh] | 45710 |
| - ambulatory care facilities[Mesh] | 44180 |
| - ambulatory medicine*[tw] | 181 |
| - ambulatory centre*[tw] | 18 |
| - ambulatory clinic*[tw] | 611 |
| - ambulatory service*[tw] | 465 |
| - ambulatory department*[tw] | 34 |
| - ambulatory room*[tw] | 2519 |
| - ambulatory ward*[tw] | 3 |
| - ambulatory unit*[tw] | 88 |
| - ambulatory treatment*[tw] | 1431 |
| - ambulatory care*[tw] | 52285 |
| - ambulatory patient*[tw] | 3957 |
| - ambulatory physician*[tw] | 85 |
| - outpatients[Mesh] | 9291 |
| - outpatient medicine*[tw] | 55 |
| - outpatient centre*[tw] | 92 |
| - outpatient clinic*[tw] | 32540 |
| - outpatient service*[tw] | 2923 |
| - outpatient department*[tw] | 4746 |
| - outpatient room*[tw] | 13 |
| - outpatient ward*[tw] | 95 |
| - outpatient unit*[tw] | 659 |
| - outpatient treatment*[tw] | 4433 |
| - outpatient care*[tw] | 3342 |
| - outpatient physician*[tw] | 205 |
| - accident medicine*[tw] | 33 |
| - accident centre* [tw] | 15 |
| - accident clinic* [tw] | 6 |
| - accident service*[tw] | 90 |
| - accident department*[tw] | 106 |
| - accident room*[tw] | 7 |
| - accident ward*[tw] | 11 |
| - accident unit*[tw] | 34 |
| - accident treatment*[tw] | 21 |
| - accident care*[tw] | 12 |
| - accident patient*[tw] | 146 |
| - accident physician*[tw] | 7045 |
| - trauma medicine*[tw] | 29 |
| - trauma centre*[tw] | 1375 |
| - trauma clinic* [tw] | 184 |
| - trauma service*[tw] | 771 |
| - trauma department*[tw] | 129 |
| - trauma room*[tw] | 155 |
| - trauma ward*[tw] | 63 |
| - trauma unit*[tw] | 548 |
| - trauma treatment*[tw] | 212 |
| - trauma care*[tw] | 2700 |
| - trauma patient*[tw] | 12384 |
| - trauma physician*[tw] | 28 |
| - triage medicine[tw] | 0* |
| - triage centre*[tw] | 12 |
| - triage clinic*[tw] | 35 |
| - triage service*[tw] | 79 |
| - triage department*[tw] | 4 |
| - triage room*[tw] | 12 |
| - triage ward*[tw] | 233 |
| - triage unit*[tw] | 33 |
| - triage treatment*[tw] | 3607 |
| - triage care*[tw] | 10 |
| - triage patient*[tw] | 281 |
| - triage physician*[tw] | 10 |
| - urgent care[tw] | 1089 |
| - urgent care medicine*[tw] | 35 |
| - urgent care centre*[tw] | 25 |
| - urgent care clinic*[tw] | 120 |
| - urgent care service*[tw] | 51 |
| - urgent care department*[tw] | 13 |
| - urgent care room*[tw] | 1 |
| - urgent care unit*[tw] | 6 |
| - urgent care treatment*[tw] | 23 |
| - urgent care patient*[tw] | 8 |
| - urgent care physician*[tw] | 8 |
| - (OR/1-87) | **291775** |
| - prevalence[Mesh] | 197000 |
| - incidence[Mesh] | 180216 |
| - prevalence*[tw] | 477728 |
| - incidence*[tw] | 632904 |
| - occurrence*[tw] | 260129 |
| - commonness*[tw] | 182 |
| - frequency*[tw] | 652873 |
| - (OR/89-95) | **1622686** |
| - back pain[Mesh] | 29440 |
| - low back pain[Mesh] | 14613 |
| - sciatic neuropathy[Mesh] | 5775 |
| - dorsalgia[tw] | 64 |
| - back pain[tw] | 43975 |
| - backach*[tw] | 3398 |
| - back ach*[tw] | 80 |
| - lumbar pain*[tw] | 191 |
| - coccyx[tw] | 1229 |
| - coccydynia[tw] | 84 |
| - sciatica[tw] | 5773 |
| - spondylosis[tw] | 3292 |
| - lumbago[tw] | 1164 |
| - back disorder*[tw] | 513 |
| - (OR/97-110) | **55738** |
| - (88 AND 96 AND 111) | **263/340** |

Notes: Left column presents the MESH terms and keywords searched. Right column presents the number of studies returned from each search.
