# Supplementary material for: Prevalence of low back pain in emergency settings: a systematic review and meta-analysis
Source: BMC Musculoskelet Disord. 2017 Apr 4;18:143. doi: 10.1186/s12891-017-1511-7 (PMC5379602; doi:10.1186/s12891-017-1511-7)
Supplement: Supplementary file 2 — EMBASE Search Strategy. (DOCX 79 kb) [file 12891_2017_1511_MOESM2_ESM.docx]

# **Additional File 2: EMBASE Search Strategy**

| 1. ‘emergency health service’/exp |
| --- |
| 1. ‘emergency ward’/exp |
| 1. ‘emergency treatment’/exp |
| 1. ‘emergency care’/exp |
| 1. ‘evidence based emergency medicine’/exp |
| 1. ‘ambulatory care’/exp |
| 1. ‘outpatient’/exp |
| 1. ‘outpatient department’/exp |
| 1. ‘outpatient care’/exp |
| 1. urgicenter:ab,ti |
| 1. emergicenter:ab,ti |
| 1. ((emergenc* OR ambulatory OR outpatient* OR accident* OR urgent* OR trauma*) NEAR/2 (medicine* OR centre* OR center* OR clinic* OR service* OR department* OR room* OR ward* OR unit* OR treatment* OR care* OR patient* OR physician* OR doctor*)):ab,ti |
| 1. 1-12 OR |
| 1. ‘prevalence’/exp |
| 1. ‘incidence’/exp |
| 1. prevalence*:ab,ti OR incidence*:ab,ti OR occurrence*:ab,ti OR commonness:ab,ti OR frequency*:ab,ti |
| 1. 14-16 OR |
| 1. ‘low back pain’/exp |
| 1. ‘backache’/exp |
| 1. ‘sciatic neuropathy’/exp |
| 1. dorsalgia:ab,ti |
| 1. backache:ab,ti |
| 1. coccyx:ab,ti |
| 1. coccydynia:ab,ti |
| 1. sciatica:ab,ti |
| 1. spondylosis:ab,ti |
| 1. lumbago:ab,ti |
| 1. ((back OR lumbar) NEAR/2 pain*):ab,ti |
| 1. (back NEAR/2 disorder*):ab,ti |
| 1. 18-29 OR |
| 1. 13 AND 17 AND 30 **683/902** |

Notes: Presents search terms and final number of studies returned from the final search.
