# Supplementary material for: Prevalence of low back pain in emergency settings: a systematic review and meta-analysis
Source: BMC Musculoskelet Disord. 2017 Apr 4;18:143. doi: 10.1186/s12891-017-1511-7 (PMC5379602; doi:10.1186/s12891-017-1511-7)
Supplement: Supplementary file 3 — Grey Literature Search Strategy. (DOCX 54 kb) [file 12891_2017_1511_MOESM3_ESM.docx]

# **Additional File 3: Grey Literature Search Strategy**

| **Included Websites from Grey Matters** | **Titles Searched** |
| --- | --- |
| **Health Economics** | |
| Health Agency of Canada (PHAC) | 100 |
| Agency for Healthcare Research and Quality National Quality Measured Clearinghouse. | 200 |
| **Health Statistics** | |
| Canadian Institute for Health Information (CIHI) | 100 |
| Health Canada | 100 |
| Institute for Clinical Evaluation Sciences | 250 |
| Institute of Health Economics | 2 |
| PHAC Reports and Publications and Surveillance | 100 |
| Stats Canada | - |
| CDC National Center for Health Statistics | 250 |
| WHO | 100 |
