# Supplementary material for: Prevalence of low back pain in emergency settings: a systematic review and meta-analysis
Source: BMC Musculoskelet Disord. 2017 Apr 4;18:143. doi: 10.1186/s12891-017-1511-7 (PMC5379602; doi:10.1186/s12891-017-1511-7)
Supplement: Supplementary file 4 — Data Extraction Form. (DOCX 107 kb) [file 12891_2017_1511_MOESM4_ESM.docx]

# **Additional File 4: Data Extraction Form**

| **Study Title** |
| --- |
| **Primary Author** |
| **Publication Year** |
| **Country /Region** |
| **Study Objectives** |
| **Study Design** |
| **Data Source *(Description)*** |
| **Data Source *(Category)*** |
| **Study Duration (Timeline)** |
| **Setting Service *(Description)*** |
| **Setting Service *(Category)*** *(*Admin data, Survey Data, Patient Charts, Other) |
| **Setting Health Care System *(Description)*** |
| **Setting Health Care System *(Category)*** *(EMS, ED, Hospital, EMS and ED, EMS and Hospital, ED and Hospital, EMS and ED and Hospital, Community ED Centre.* |
| **Population *(Description)*** |
| **Population *(Category)*** *(Metropolitan, Rural, Both, Other)* |
| **Case Definition (Overall, Anatomy, Duration, Signs/Symptoms, Activity Limits, Anything Non-Medical)** |
| **Inclusion Criteria** |
| **Exclusion Criteria** |
| **Age (Range)** |
| **Age (Mean)** |
| **Sex** |
| **Workers Compensation** |
| **Week Days Presenting** |
| **Classification of LBP *(Description)*** |
| **Classification of LBP *(Category)*** *(Non-specific, Muscular, Non-Muscular, All Back Pain, Other)* |
| **LBP Coding System *(Descriptive)*** |
| **LBP Coding System *(Category)*** *(EDIS Triage Coding, ICD-9, ICD-10, Clinical classification software, Canadian Emergency Department Diagnosis Shortlist, Other)* |
| **Assignment of LBP Code *(Descriptive)*** |
| **Assignment of LBP Code *(Category)*** *(Paramedic, Triage Nurse, Nurse, Physician, Other)* |
| **LBP Code Limits (Codes Used to Define Back Pain)** |
| **Stratification / Standardization *(Descriptive)*** |
| **Stratification *(Category)*** *(Age, Sex, Location, Income, Workers Compensation, None, Other)* |
| **Sample Size** |
| **Prevalence (%)** |
| **Authors Conclusions / Outcome** |
| **Limitations: data entered by the people, Muscular and non-muscular** |
