# Supplementary material for: Prevalence of low back pain in emergency settings: a systematic review and meta-analysis
Source: BMC Musculoskelet Disord. 2017 Apr 4;18:143. doi: 10.1186/s12891-017-1511-7 (PMC5379602; doi:10.1186/s12891-017-1511-7)
Supplement: Supplementary file 5 — Risk of Bias Tool Developed By Hoy et al., [3]. (DOCX 69 kb) [file 12891_2017_1511_MOESM5_ESM.docx]

# **Additional File 5: Risk of Bias Tool Developed By Hoy et al., 2012.**

| External Validity  1.Was the sampling frame a true or close representation of the target population?  2. Was some form of random selection used to select the sample, OR was a census undertaken?  3. Was the likelihood of nonresponse bias minimal? |
| --- |
| **Internal Validity**  4. Were data collected directly from the subjects (as opposed to a proxy)?  5. Was an acceptable case definition used in the study?  6. Was the study instrument that measured the parameter of interest shown to have validity and reliability?  7. Was the same mode of data collection used for all subjects?  8. Was the length of the shortest prevalence period for the parameter of interest appropriate?  9. Were the numerator(s) and denominator(s) for the parameter of interest appropriate?  10. Summary item on the overall risk of study bias (?). |

Notes: The modified tool assesses each study according to nine domains: three external validity domains, and six internal validity domains, plus one item assessing overall risk of bias. The external validity domains assess the target population; sampling and non-response bias, while the internal risk of bias domains assess data collection, case definitions, assessment tools, prevalence period and an assessment of the numerator and denominator. We modified the original tool by omitting an additional domain that assesses whether the study population represents the national population, which was not relevant to our review.
