# Supplementary material for: Prevalence of low back pain in emergency settings: a systematic review and meta-analysis
Source: BMC Musculoskelet Disord. 2017 Apr 4;18:143. doi: 10.1186/s12891-017-1511-7 (PMC5379602; doi:10.1186/s12891-017-1511-7)
Supplement: Supplementary file 6 — GRADE Concepts Developed by Guyatte et al., 2011. (DOCX 75 kb) [file 12891_2017_1511_MOESM6_ESM.docx]

# **Additional File 6: GRADE Concepts Developed by Guyatte et al., 2011.**

| **Evidence about Prevalence** |
| --- |
| **Study Limitations:** Serious limitations when most evidence is from studies with moderate or unclear risk of bias for most bias domains. Very Serious limitations when most evidence is from studies with high risk of bias for almost all bias domains. |
| **Inconsistency:** Unexplained heterogeneity or variability in results across studies with differences of results not clinically meaningful. For narrative summary: variations in prevalence estimates across studies. |
| **Indirectness:** The study sample, and the outcome or prevalence estimate in the primary studies do not accurately reflect the review question. Generalizability of the study population (is the study population a subset of the population of interest?). |
| **Imprecision:** For narrative summary: within-study imprecision: sample size justification is not provided for prevalence estimates. Across study imprecision: there are few studies and small number of participants across studies. |
| **Publication Bias:** Published evidence is restricted to only a portion of the studies conducted on the topic. |

Notes: Adapted components of the GRADE framework to assess the overall quality of the available evidence on the prevalence of low back pain in the emergency setting ^(59)^.
